# Preoperative determinants of quality of life a year after coronary artery bypass grafting: a historical cohort study

**DOI:** 10.1186/s13019-018-0798-2

**Published:** 2018-11-19

**Authors:** Lisa Verwijmeren, Peter Gerben Noordzij, Edgar Jozeph Daeter, Bas van Zaane, Linda Margaretha Peelen, Eric Paulus Adrianus van Dongen

**Affiliations:** 1 Anesthesiology, Intensive Care and Pain Medicine, St. Antonius Hospital, Koekoekslaan 1, Nieuwegein, 3430 EM The Netherlands; 2 Cardiac Surgery, St. Antonius Hospital, Koekoekslaan 1, Nieuwegein, 3430 EM The Netherlands; 3 Anesthesiology, Intensive Care and Emergency Medicine, University Medical Center Utrecht, Utrecht University, Heidelberglaan 100, Utrecht, 3584 CX The Netherlands; 4 Epidemiology, Julius Center for Health Sciences and Primary Care, University Medical Center Utrecht, Utrecht University, Heidelberglaan 100, Utrecht, 3584 CX The Netherlands

**Keywords:** Quality of life, Coronary artery bypass graft surgery, Risk factors

## Abstract

**Background:**

Health related quality of life (HRQL) is an important patient related outcome measure after cardiac surgery. Preoperative determinants for postoperative HRQL have not yet been identified, but could aid in preoperative decision making. The aim of this article is to identify associations between preoperative determinants and change in HRQL 1 year after coronary artery bypass grafting (CABG).

**Methods:**

Single centre retrospective cohort study in 658 patients. Change in HRQL was defined as a decrease or increase of ≥5 points on the physical or mental domain of the Short Form 12 (SF-12) questionnaire. Patients were stratified in three groups according to worse, unchanged, or better HRQL. Multinomial logistic regression analysis was used to investigate the association between preoperative risk factors and postoperative change in HRQL.

**Results:**

Physical HRQL improved in 22.8% of patients, did not change in 61.2% of patients and worsened in 16.0% of patients. Comorbidities associated with change in physical HRQL were a history of stroke, atrial fibrillation, vascular disease or pulmonary disease. Most important risk factor for change in physical HRQL was preoperative HRQL. Higher preoperative SF-12 score decreased the odds for worse physical HRQL and increased the odds for better physical HRQL. Mental HRQL improved in 49.8% of patients, remained unchanged in 34.5% of patients and worsened in 15.7% of patients. Preoperative HRQL was an important risk factor for a change in mental HRQL. Higher preoperative physical HRQL increased the odds for improved mental HRQL. Lower preoperative mental HRQL increased the odds for better mental HRQL.

**Conclusions:**

One year after CABG the majority of patients experiences equal or improved HRQL compared to before surgery. Most important preoperative risk factor for change in HRQL is preoperative HRQL.

**Electronic supplementary material:**

The online version of this article (10.1186/s13019-018-0798-2) contains supplementary material, which is available to authorized users.

## Background

In the past years the population of patients referred for coronary artery bypass grafting (CABG) changed to an older and more complex population [1–3]. Assumed causes are an increased life expectancy and improvements in surgical and anaesthetic techniques, making it possible for elderly high risk patients to undergo surgery [1, 2]. Even though CABG is considered safe for elderly patients, a considerable risk for complications or mortality remains [4]. Especially in more complex patients a tailored approach is needed in which health benefits are weighed against risk of complications. Risk stratification tools like the European System for Cardiac Operative Risk Evaluation (euroSCORE), Parsonnet score or the American College of Surgeons Risk Calculator can aid in estimating outcome after surgery [5–7]. However, these tools were developed to predict morbidity or mortality, not health related quality of life (HRQL) [8]. In general, HRQL improves after cardiac surgery, but it is known that 8–19% of patients experiences a decrease in HRQL [9]. In addition to the traditional outcome parameters of major morbidity or mortality, information on patient-perceived postoperative HRQL is crucial for full informed consent prior to surgery. To optimize preoperative risk-assessment and facilitate shared decision making, more accurate data on risk factors that influence postoperative HRQL are needed. This study aims to identify which preoperative determinants are associated with a clinically relevant change in HRQL 1 year after CABG.

## Materials and methods

### Design

This was a single centre retrospective cohort study. Since patients were not subjected to investigational actions and were treated according to standard guidelines the need for informed consent was waived by the local review board of the ethical committee (Medical research Ethics Committee United, number W15.069). The study was conducted in accordance with the principles of the Declaration of Helsinki.

### Population

All patients older than 18 years who underwent elective isolated CABG between the first of July 2011 and the first of May 2014 were eligible for inclusion. Inclusion criteria were a completed medical outcomes study Short Form (SF) 12 version 2 questionnaire at baseline and at 12 months after CABG. Surgical procedures were performed by multiple cardiothoracic surgery consultants and their supervised trainees in a tertiary referral hospital (St. Antonius Hospital, Nieuwegein, The Netherlands). Perioperative care was carried out according to standard clinical practice and based on international guidelines for all patients [10].

### Clinical characteristics and data collection

As potential determinants for postoperative HRQL patient characteristics, medical history and comorbidities, preoperative laboratory tests and preoperative HRQL were considered.

Data on age, gender, body mass index and preoperative laboratory tests were collected from the routine preoperative anaesthesia visit. Medical history, additive euroSCORE and postoperative complications were collected from the hospital’s electronic database for cardiac surgery patients. Registration of postoperative complications was conducted in the context of a national registry of cardiac interventions in The Netherlands (Supervisory Committee for Cardiac Interventions (Begeleidingscommissie Hartinterventies Nederland) [11]. Intraoperative data was collected from the computerized medical record (MetaVision 5.46.44, iMDsoft®, Düsseldorf, Germany). A manual review of the data was performed to check for accuracy and missing values by a member of the research team (LV). Missing data on laboratory values, medication use and surgical characteristics were fully completed by manually checking electronic patient records.

#### Outcomes

Primary outcome was change in HRQL 12 months after surgery measured with the SF-12 before surgery and at 1 year after surgery. The SF-12 is derived from the SF-36, a widely validated questionnaire including 36 questions on physical and mental well-being [12]. The SF-12 is a validated shorter version with 12 questions [13]. Questions include, but are not limited to items relating to overall health perception, being able to exercise, feeling at ease or feeling full of energy. Answers on these 12 items are converted into two norm-based scores ranging from 0 to 100, representing physical and mental HRQL [14]. Higher scores represent better HRQL. Additional file 1: Table S1 presents reference scores for the Dutch population.

Secondary outcome was a composite endpoint of any complication and defined as one or more of the following; new atrial fibrillation defined as rhythm disturbance requiring treatment including reanimation, defibrillation or medication; re-sternotomy, defined as reoperation requiring opening of the sternum; myocardial infarction, defined as presence of ≥2 of the following symptoms: prolonged typical chest pain, ≥ten-fold increase of cardiac enzyme levels, and new wall motion abnormalities and/or changes in two or more leads in at least two consecutive ECGs; deep sternal wound infection, defined as requiring surgical drainage or fixation, positive wound cultures or antibiotic therapy; and ischemic stroke, defined as an acute episode of cerebral, spinal or retinal dysfunction caused by infarction of the central nervous system lasting > 72 h.

#### Procedures

As part of routine care, SF-12 questionnaires were sent by mail to all patients before surgery and were collected at the time of hospital admission. Twelve months after the operation a questionnaire was sent by email to each surviving patient. This study was carried out as a retrospective analysis of data collected for quality assessment purposes. In this process no second request was made to retrieve a missing questionnaire when a patient did not respond.

### Statistical analysis

Data are presented as frequencies and percentages of total for categorical data, as mean ± standard deviation (SD) for normally distributed continuous data and as median and interquartile range (IQR) for non-normally distributed continuous data. Normality was tested using visual inspection of histograms and Kolmogorov-Smirnov test. Scores for preoperative HRQL were compared to the Dutch population mean using a one sample t-test. A paired sample t-test was used to compare preoperative HRQL to scores at 1 year after surgery. A delta score was calculated for change in HRQL by subtracting preoperative SF-12 scores from postoperative SF-12 scores. A positive delta score represents an improvement in HRQL.

To study the clinical relevance of the difference in HRQL a threshold value was used to compare groups. As standard threshold values are lacking in CABG surgery for the SF-12 questionnaire, we based our threshold value on two articles using the SF-36 in elderly patients undergoing CABG or aortic valve surgery. Welke et al. defined a clinically relevant change as a difference of ≥5.42 points in physical or ≥ 6.33 in mental HRQL [15]. Jansen-Klomp et al. used a cut-off point of ≥2.5 points [16]. As with the SF-12 questionnaire the HRQL could increase or decrease as much as four points by changing the results of a single question, we set the threshold value at 5 or more points decrease or increase to ensure a clinically relevant change. Subsequently, the study cohort was stratified in three groups according to change in HRQL: worse, no change or better HRQL. Differences between the three groups were tested using Chi square test for dichotomous or categorical variables and one way ANOVA or Kruskal Wallis test for continuous data depending on normality.

To analyse the independent effects of all risk factors a multivariable model was built using multinomial logistic regression analysis. This analysis allows for a dependent variable with more than two categories. Patients with no clinically relevant change in HRQL were used as reference category to which the outcomes ‘worse HRQL’ and ‘better HRQL’ were compared. No variable selection took place and all variables were added simultaneously. To prevent multicollinearity, correlations between all variables were tested using Pearson’s correlation coefficient. Of variables with a correlation > 0.8 one variable was excluded from the model. Non-linearity of the continuous variables regarding preoperative physical and mental HRQL was investigated by adding various transformations and assessing model fit in terms of log likelihood. If model fit improved, the transformations were retained in the multivariable model. Results are presented as odds ratios with their accompanying confidence intervals. For statistical analysis IBM SPSS version 22 (IBM Corp. Released 2013. IBM SPSS Statistics for Windows, Version 22.0. Armonk, NY: IBM Corp.) was used. A *p*-value < 0.05 was considered as statistically significant for all analyses.

## Results

### Study population

**Fig. 1:**
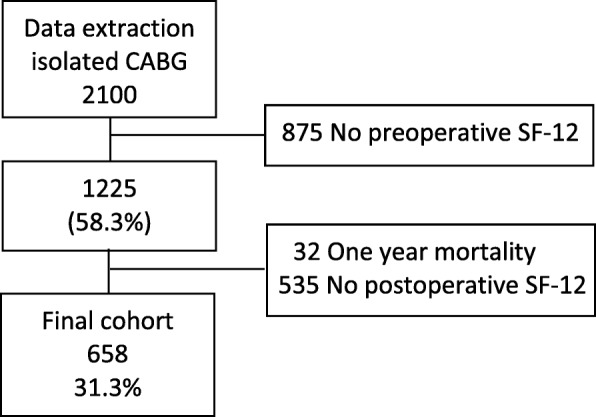
Flowchart of patient selection

In total, 2100 patients underwent elective isolated CABG. Of these patients, 1225 completed a baseline SF-12. Thirty-two patients died within 1 year. 658 (31.3% of total, 53.7% of 1225) patients returned the 12 month follow up questionnaire and were included in the final cohort (Fig. 1). Overall, non-responders had more comorbidities compared to responders. This was expressed by significant differences in age, incidence of male gender, diabetes mellitus, prior myocardial infarction, LVEF and euroSCORE. Additional file 2: Table S2 shows other baseline variables for responders and non-responders.

### Patient characteristics and outcome

Mean age was 66 years (SD ±9) minimum age was 47 and maximum age was 87 years old. The majority of patients was male (82.4%). Baseline characteristics including preoperative HRQL are presented in Table 1. In-hospital complications occurred in 41 (6.2%) patients. Postoperative atrial fibrillation was most common and occurred in 19 (2.9%) patients. Median length of stay was 6 (IQR 4–7) days. Mean preoperative physical HRQL in the study cohort was lower compared to the Dutch population mean (39.2 (SD ±5.4) vs 50.6 (SD ±9.2) *p* < 0.001 respectively, difference: -11.4; 95% CI -11.8 to − 11.0). One year after surgery mean physical HRQL increased slightly to 39.9 (SD ±4.6) (*p* = 0.007) but remained below the population mean (difference − 10.7; 95% CI -11.0 to − 10.3). Mean preoperative mental HRQL was lower compared to the population mean (48.9 (SD ±13.1) vs 50.2 (SD ±9.2) *p* = 0.001 respectively, difference: -1.7; 95% CI -2.7 to − 0.7). Mean mental HRQL improved to 54.4 (SD ±12.6) (*p* < 0.001) after surgery and rose above population mean (difference 3.8; 95% CI 2.8 to 4.8).

**Table 1:** Baseline

	*N* = 658
Age (years)	65.5 ± 9.0
Male gender	542 (82.4%)
Current smoking	191 (29.0%)
Body mass index (kg/m2)	26.8 (24.6–29.8)
Hypertension	338 (51.4%)
Myocardial infarction	75 (11.4%)
Stroke	54 (8.2%)
Pulmonary disease	50 (7.6%)
Atrial fibrillation	26 (4.0%)
Peripheral vascular disease	68 (10.3%)
Unstable angina	65 (9.9%)
Diabetes mellitus	133 (20.2%)
Left ventricular ejection fraction	
Good (≥50%)	546 (83.0%)
Moderate (30–50%)	94 (14.3%)
Poor (< 30%)	18 (2.7%)
Additive EuroSCORE	3 (1–4)
Preoperative hemoglobine (mmol/l)	8.8 (8.2–9.3)
Preoperative creatinine (μmol/)	82 (73–95)
Health related quality of life	
Preoperative physical HRQL	39.2 ± 5.4 39.0 (36.0–42.6)
Preoperative mental HRQL	48.9 ± 13.1 50.3 (40.2–59.0)
Surgical characteristics	
Duration of surgery (min)	189 (163–226)
Extra corporeal circulation time(min)	83 (68–101)
Mini extra corporeal circulation	485 (73.7%)
Use of internal mammary artery	626 (95.1%)
Packed red blood cell transfusion^a^	54 (8.2%)
Blood loss (ml)^b^	660 (519–850)

Data are presented as mean (±standard deviation), medians (interquartile range) or frequencies (%)

*HRQL* health related quality of life

^a^Intraoperative packed red blood cell transfusion

^b^Blood loss at 24 h after surgery

### Determinants of a clinically relevant change in physical HRQL

Physical HRQL 1 year after surgery improved in 150 (22.8%) patients, did not change in 403 (61.2%) patients and got worse in 105 (16.0%) patients. Preoperative median EuroSCORE was 3 (IQR 1–4), 3 (IQR 1–4), and 3 (IQR 1–5) for worse, no change or better physical HRQL respectively (*p* = 0.802). Median preoperative physical HRQL was 45.3 (IQR 41.5–48.5) for patients with worse physical HRQL; 39.7 (IQR 37.1–42.1) for patients with no change in physical HRQL and 34.8 (IQR 31.3–37.2) for patients with better HRQL (*p* < 0.001). Median preoperative mental HRQL was 47.2 (IQR 35.9–54.1); 50.5 (IQR 40.8–59.2) and 52.6 (IQR 43.1–60.8) for patients with worse, no change or better HRQL respectively (*p* = 0.001). Postoperative complications occurred in 6.7% of patients with worse physical HRQL, in 5.5% of patients with no change in physical HRQL and in 8.0% of patients with better physical HRQL (*p* = 0.536).

Table 2 shows the independent odds ratios of preoperative risk factors for a change in physical HRQL. Clinical features significantly associated with a change in physical HRQL were a history of stroke, atrial fibrillation, vascular disease or pulmonary disease. Patients with a prior stroke showed increased odds for worse physical HRQL. A history of atrial fibrillation or vascular disease decreased the odds for a worse physical HRQL and a history of pulmonary disease increased the odds for improved HRQL. Preoperative physical HRQL was significantly associated with a change in physical HRQL. A higher preoperative value on the SF-12 decreased the odds for worse HRQL and increased the odds for better HRQL.

**Table 2:** Multinomial regression analysis for change in physical HRQL

	Worse physical HRQL		Better physical HRQL	
	Beta	OR (95% CI)	Beta	OR (95% CI)
Age (per year)	−0.03	0.97 (0.92–1.01)	−0.00	1.00 (0.95–1.04)
Body mass index (per point)	0.02	1.02 (0.94–1.09)	− 0.04	0.96 (0.91–1.02)
EuroSCORE (per point)	0.16	1.18 (0.95–1.46)	−0.03	0.98 (0.81–1.18)
LVEF > 50%	0	1.00	0	0
LVEF 30–50%	1.67	5.32 (0.41–69.61)	0.60	1.82 (0.43–7.65)
LVEF < 30%	1.15	3.16 (0.25–39.56)	0.66	1.94 (0.50–7.61)
Hemoglobin (per point)	− 0.03	0.97 (0.65–1.44)	− 0.24	0.79 (0.56–1.10)
Creatinine (per point)	− 0.01	0.99 (0.98–1.01)	− 0.00	1.00 (0.99–1.01)
Preoperative physical HRQL (per point)				
Singular	−1.25^***^		0.54	
Quadratic	0.02^***^	^a^	−0.01^*^	^a^
Preoperative mental HRQL (per point)	0.00	1.00 (0.98–1.03)	0.01	1.01 (0.99–1.03)
Male gender	0.07	1.07 (0.43–4.47)	0.11	1.12 (0.52–2.41)
Active smoking	0.41	1.51 (0.79–2.88)	0.45	1.57 (0.91–2.69)
Hypertension	0.51	1.67 (0.91–3.06)	− 0.04	0.96 (0.60–1.55)
Myocardial infarction	0.18	1.19 (0.50–2.84)	0.37	1.45 (0.69–3.04)
Stroke	0.99^*^	2.68 (1.04–6.88)	0.47	1.60 (0.98–3.79)
Pulmonary disease	0.44	1.56 (0.55–4.43)	0.86^*^	2.36 (1.05–5.31)
Atrial fibrillation	−3.17^*^	0.04 (0.00–0.97)	−1.50	0.22 (0.05–1.10)
Peripheral vascular disease	− 1.23^*^	0.29 (0.10–0.90)	0.20	1.23 (0.57–2.64)
Unstable angina	−0.47	0.62 (0.24–1.63)	0.22	1.25 (0.57–2.75)
Diabetes mellitus	0.22	0.25 (0.59–2.65)	−0.13	0.88 (0.49–1.60)

Unchanged HRQL was the reference category. Worse and Better HRQL were defined as ≥5 points change in delta HRQL

*LVEF* left ventricular ejection fraction, *HRQL* health related quality of life

^*^ = *p* < 0.050, ^***^ = *p* < 0.001

^a^Since clinical interpretation of odds ratios is limited for quadratic equations this is not presented

### Determinants of a clinically relevant change in mental HRQL

Mental HRQL improved in 328 (49.8%) patients at 1 year after surgery, remained unchanged in 227 (34.5%) patients and worsened in 103 (15.7%) patients. Preoperative median euroSCORE was 3 (IQR 1–5) for patients with worse mental HRQL, 3 (IQR 1–4) for patients with no change in mental HRQL, and 3 (IQR 1–4) for patients with better mental HRQL (*p* = 0.887). Prior to surgery, physical HRQL was 37.9 (IQR 35.0–41.7); 37.9 (IQR 35.1–41.4) and 40.5 (IQR 37.0–43.9) for patients with worse, no change or better HRQL respectively (*p* < 0.001). Preoperative mental HRQL was 55.2 (IQR 46.9–63.7); 55.6 (IQR 46.6–63.6) and 45.1 (IQR 34.7–53.4) for patients with worse, no change or better HRQL respectively (*p* < 0.001). Postoperative complications were seen in 8.7% of patients with worse mental HRQL, in 5.3% of patients with no change in mental HRQL and in 6.1% of patients with better mental HRQL (*p* = 0.481).

Independent odds ratios of preoperative risk factors for a change in mental HRQL are shown in Table 3. No significant associations were seen between clinical risk factors and worse or better mental HRQL 1 year after CABG. Higher preoperative physical HRQL increased the odds for improved mental HRQL. Lower preoperative mental HRQL increased the odds for better mental HRQL.

**Table 3:** Multinomial regression analysis for change in mental HRQL

	Worse mental HRQL		Better mental HRQL	
	Beta	OR (95% CI)	Beta	OR (95% CI)
Age (per year)	−0.02	0.98 (0.93–1.02)	0.01	1.01 (0.97–1.04)
Body mass index (per point)	0.02	1.02 (0.96–1.08)	0.01	1.01 (0.96–1.06)
EuroSCORE (per point)	0.02	1.02 (0.85–1.23)	−0.07	0.94 (0.80–1.10)
LVEF > 50%	0	0	0	0
LVEF 30–50%	−0.36	0.84 (0.21–3.39)	1.13	3.09 (0.60–15.83)
LVEF < 30%	− 0.18	0.70 (0.19–2.63)	0.66	1.93 (0.40–9.36)
Hemoglobin (per point)	−0.15	0.86 (0.61–1.23)	0.03	1.07 (0.80–1.43)
Creatinine (per point)	0.01	1.01 (1.00–1.02)	−0.00	1.00 (0.99–1.01)
Preoperative physical HRQL (per point)	− 0.00	1.00 (0.95–1.05)	0.08^***^	1.08 (1.04–1.13)
Preoperative mental HRQL (per point)				
Singular	0.02		−1.07^**^	
Quadratic	−0.01		0.02^**^	
Cubic	0.00	^a^	0.00^**^	^a^
Male gender	0.15	1.17 (0.52–2.61)	−0.04	0.96 (0.50–1.85)
Active smoking	− 0.09	0.92 (0.51–1.65)	0.03	1.04 (0.65–1.65)
Hypertension	0.16	1.18 (0.71–1.95)	0.12	1.13 (0.75–1.71)
Myocardial infarction	0.20	1.23 (0.60–2.51)	−0.56	0.57 (0.29–1.11)
Stroke	− 0.03	0.97 (0.37–2.50)	0.18	1.19 (0.57–2.52)
Pulmonary disease	0.37	1.45 (0.63–3.34)	−0.62	0.54 (0.25–1.14)
Atrial fibrillation	0.39	1.48 (0.44–4.99)	−0.35	0.71 (0.23–2.15)
Peripheral vascular disease	0.00	1.00 (0.41–2.44)	0.34	1.41 (0.72–2.77)
Unstable angina	0.17	1.12 (0.52–2.74)	0.20	1.22 (0.62–2.41)
Diabetes mellitus	− 0.6	0.94 (0.49–1.81)	0.02	1.02 (0.60–1.74)

Unchanged HRQL was the reference category. Worse and Better HRQL were defined as ≥5 points change in delta HRQL respectively

*LVEF* Left ventricular ejection fraction, *HRQL* health related quality of life

^**^p = < 0.005, ^***^ = *p* < 0.00

^a^Since clinical interpretation of odds ratios is limited for quadratic and cubic equations this is not presented

## Discussion

In this study the most important determinant for a change in HRQL 1 year after CABG was preoperative HRQL. Higher preoperative physical HRQL led to improved outcomes regarding physical and mental HRQL at 1 year after surgery considering other clinical risk factors. Lower mental HRQL before surgery increased the chance to improve in mental HRQL at 1 year after surgery. The influence of preoperative HRQL on a change after surgery illustrates the vital importance of acquiring information on HRQL in the preoperative setting in order to fully inform patients on expected patient-centred outcomes.

### Change in HRQL

In the majority of patients physical HRQL hardly changed, as was reflected by a mean increase of 0.7 points. Mental HRQL increased in half of patients, with a mean increase of 5.5 points.

Contrasting to our findings, other studies using the SF-36, showed a mean increase in physical HRQL ranging from 4.8 to 5.3 points and a mean increase in mental HRQL of 1.2 to 1.9 points [17, 18]. In a cohort of 1744 patients, Rumsfeld et al. assessed SF-36 before and 6 months after CABG surgery. Health related quality of life increased 5.3 and 1.9 points for physical and mental HRQL respectively. Comparable to our results, preoperative physical HRQL was identified as the most important risk factor for a change in HRQL after taking other preoperative cardiac and non-cardiac risk factors into account [17].

Deutsch et al. performed a study in 106 octogenarians undergoing CABG, valve surgery or CABG combined with valve surgery. They assessed HRQL by SF-36 three and 12 months after surgery and compared it to preoperative scores. At 3 months physical HRQL significantly increased with 5.1 points and mental HRQL was comparable to preoperative levels. Cardiac and non-cardiac comorbidities and procedural data were not identified as relevant risk factors for change in HRQL. Unfortunately, baseline HRQL was not considered as possible risk factor for a change in HRQL in their study [18].

In contrast to other literature reports, physical HRQL at 1 year after surgery increased to a lesser extent in our study, while the increase in mental HRQL was more eminent [15, 17, 18].

These differences could be due to the moment of measuring HRQL. At 1 year after surgery, which was the moment of measurement in our study, HRQL could have been affected by other factors as well, resulting in lower scores.

Furthermore, CABG is mainly performed to relieve complaints of angina, which is likely to result in improved physical functioning. In our cohort less than 10% of patients suffered from unstable angina while this was present in up to 28–61% in patients in other articles [17, 19]. It is conceivable that patients in this study suffered from fewer complaints before surgery and therefore did not notice a relevant increase in physical HRQL. Also, questions regarding mental HRQL in the SF-12 include items as feeling full of energy, calm and peaceful or feeling downhearted. Although patients did not report an improvement in questions on physical functioning, CABG surgery may have improved mental status by relieving anxiety and enhancing feelings of security and self-esteem. The increase in mental HRQL in our study could reflect the overall benefit of the surgery.

### Risk factors for change in HRQL after cardiac surgery

Preoperative risk stratification based on patient-centered outcomes, such as HRQL, could have great additional value in cardiac surgery but remains challenging as well designed risk models are lacking. Possible risk factors for change in HRQL that are readily available such as comorbidities, laboratory values or LVEF have been considered by others but resulted in conflicting results. Female gender [20, 21], older age [22], diabetes mellitus [15, 21, 23], body mass index > 35 [15], low LVEF [21], pulmonary disease [15], vascular disease [15], EuroSCORE > 3 [8], deprived socio-economic status [23] and smoking [23] have been associated with worse HRQL following cardiac surgery. Older age [15], high social support [23], and EuroSCORE > 6 [24] have been associated with better HRQL after cardiac surgery. However, several other studies, including ours, found no association between preoperative clinical factors and change in postoperative HRQL [17, 19]. Studies that included preoperative HRQL in their analysis concluded that HRQL prior to surgery was the most promising predictor for postoperative change in HRQL [15, 17, 19] and that routine preoperative assessment of HRQL should be incorporated in standard care to supplement current risk assessment [25, 26].

Some limitations should be addressed. First, the retrospective design limited the amount of available data. Comparison of responders versus non-responders showed that non-responders were older, more often male and showed a higher prevalence of diabetes mellitus, myocardial infarction, lower LVEF and higher euroSCORE. These factors can have a negative effect on postoperative HRQL [15, 20–23]. With inclusion of these patients likely greater differences in HRQL might have been present and possibly more preoperative predictors would have been identified. A possible reason for non-responding could be the method of approach during the follow up period, where questionnaires were sent by email without a reminder for unanswered questionnaires. Not all patients have email, which is more often the case for elderly. Second, obviously no SF-12 scores were available for deceased patients and these patients were excluded from the analysis. Mortality risk is highest for patients with more comorbidities. It is conceivable that this excluded group of patients had more comorbidities, leading to lower scores for preoperative HRQL and, subsequently different change scores. However, 1 year mortality was merely 2.6% and it seems unlikely this had a major influence on results. Third, only elective surgery patients were analysed limiting the generalisability and excluding patients with emergency CABG. However, the indication for emergency surgery is focussed on survival, while the main indication for elective CABG is to relieve angina. In elderly patients scheduled for elective surgery risk factors for postoperative HRQL are more essential for the decision making process than in patient presenting for emergency surgery.

## Conclusion

In conclusion, 1 year after CABG surgery the majority of patients experiences equal or improved HRQL when compared to before surgery. Most important preoperative determinant for a change in HRQL is HRQL prior to surgery.

## Additional files

Additional file 1: **Table S1.** Reference scores for SF-12 health related quality of life. Table S1 shows reference scores for the Short Form 12 health related quality of life questionnaire in the Dutch population. (PDF 84 kb)

Additional file 2: **Table S2.** Baseline characteristics for patients with and without postoperative SF-12. Data are presented as mean (±standard deviation), medians (interquartile range) or frequencies (%). * Intraoperative packed red blood cell transfusion ** Blood loss at 24 h after surgery. Table S2 shows baseline characteristics for excluded patients with missing postoperative Short Form 12 questionnaires. (PDF 32 kb)

## Abbreviations

CABG: Coronary artery bypass grafting
euroSCORE: European System for Cardiac Operative Risk Evaluation
HRQL: Health related quality of life
IQR: Interquartile range
LVEF: Left ventricular ejection fraction
OR: Odds ratio
SD: Standard deviation
SF-12: Short Form 12

## References

[1] Pierri MD, Capestro F, Zingaro C, Torracca L (2010). The changing face of cardiac surgery patients: an insight into a Mediterranean region. Eur J Cardiothorac Surg.

[2] Buth KJ, Gainer RA, Legare J-F, Hirsch GM (2014). The changing face of cardiac surgery: practice patterns and outcomes 2001-2010. Can J Cardiol.

[3] Cloin ECW, Noyez L (2005). Changing profile of elderly patients undergoing coronary bypass surgery. Neth Heart J.

[4] Barsoum EA, Azab B, Patel N, Spagnola J, Shariff MA, Kaleem U (2016). Long-term outcome after percutaneous coronary intervention compared with minimally invasive coronary artery bypass surgery in the elderly. Open Cardiovasc Med J.

[5] Nashef SAM, Roques F, Michel P, Gauducheau E, Lemeshow S, Salamon R (1999). European system for cardiac operative risk evaluation (EuroSCORE). Eur J Cardiothorac Surg.

[6] Parsonnet V, Dean D, Bernstein AD (1989). A method of uniform stratification of risk for evaluating the results of surgery in acquired adult heart disease. Circulation.

[7] Bilmoria KY, Liu Y, Paruch JL, Zhou L, Kmiecik TE, Ko CYC (2014). Surgical risk calculator : a decision aide and informed consent tool for patients and surgeons. J Am Coll Surg.

[8] Loponen P, Luther M, Nissinen J, Wistbacka J-O, Biancari F, Laurikka J (2008). EuroSCORE predicts health-related quality of life after coronary artery bypass grafting. Interact Cardiovasc Thorac Surg.

[9] Abah U, Dunne M, Cook A, Hoole S, Brayne C, Vale L (2015). Does quality of life improve in octogenarians following cardiac surgery? A systematic review. BMJ Open.

[10] Wijns W, Kolh P, Danchin N, Di Mario C, Falk V, Folliguet T (2010). Guidelines on myocardial revascularization. Eur Heart J.

[11] Supervisory Committee for Cardiac Interventions in The Netherlands (Begeleidingscommissie Hartinterventies Nederland, BHN) [Internet]. Geraadpleegd van: https://nederlandsehartregistratie.nl/handboeken/. [geciteerd 2 Juni 2017]

[12] Gandek B, Ware JE, Aaronson NK, Apolone G, Bjorner JB, Brazier JE (1998). Cross-validation of item selection and scoring for the SF-12 health survey in nine countries: results from the IQOLA project. J Clin Epidemiol.

[13] De Smedt D, Clays E, Annemans L, Doyle F, Kotseva K, Pająk A (2013). Health related quality of life in coronary patients and its association with their cardiovascular risk profile: results from the EUROASPIRE III survey. Int J Cardiol.

[14] Ware J, Kosinski M, Keller SD (1996). A 12-item short-form health survey: construction of scales and preliminary tests of reliability and validity. Med Care.

[15] Welke KF, Stevens JP, Schults WC, Nelson EC, Beggs VL, Nugent WC (2003). Patient characteristics can predict improvement in functional health after elective coronary artery bypass grafting. Ann Thorac Surg.

[16] Jansen Klomp WW, Nierich AP, Peelen LM, Brandon Bravo Bruinsma GJ, Dambrink J-HE, KGM M (2016). Survival and quality of life after surgical aortic valve replacement in octogenarians. J Cardiothorac Surg.

[17] Rumsfeld JS, Magid DJ, O’Brien M, McCarthy M, MaWhinney S, Scd (2001). Changes in health-related quality of life following coronary artery bypass graft surgery. Ann Thorac Surg.

[18] Deutsch MA, Krane M, Schneider L, Wottke M, Kornek M, Elhmidi Y (2014). Health-related quality of life and functional outcome in cardiac surgical patients aged 80 years and older: a prospective single center study. J Card Surg.

[19] Grady KL, Lee R, Subačius H, Malaisrie SC, Mcgee EC, Kruse J (2011). Improvements in health-related quality of life before and after isolated cardiac operations. Ann Thorac Surg.

[20] Kendel F, Dunkel A, Müller-Tasch T, Steinberg K, Lehmkuhl E, Hetzer R (2011). Gender differences in health-related quality of life after coronary bypass surgery: results from a 1-year follow-up in propensity-matched men and women. Psychosom Med.

[21] Peric V, Borzanovic M, Stolic R, Jovanovic A, Sovtic S, Dimkovic S (2008). Predictors of worsening of patients’ quality of life six months after coronary artery bypass surgery. J Card Surg.

[22] Järvinen O, Saarinen T, Julkunen J, Huhtala H, Tarkka MR (2003). Changes in health-related quality of life and functional capacity following coronary artery bypass graft surgery. Eur J Cardiothorac Surg.

[23] Lindsay GM, Hanlon P, Smith LN, Wheatley DJ (2000). Assessment of changes in general health status using the short-form 36 questionnaire 1 year following coronary artery bypass grafting. Eur J Cardio-Thoracic Surg.

[24] Colak Z, Segotic I, Uzun S, Mazar M, Ivancan V, Majeric-Kogler V (2008). Health related quality of life following cardiac surgery — correlation with EuroSCORE. Eur J Cardio-Thoracic Surg.

[25] Rumsfeld JS, Alexander KP, Goff DC, Graham MM, Ho PM, Masoudi FA (2013). Cardiovascular health: the importance of measuring patient-reported health status a scientific statement from the American heart association. Circulation.

[26] Spertus JA (2008). Evolving applications for patient-centered health status measures. Circulation.

