# Supplementary material for: Preoperative determinants of quality of life a year after coronary artery bypass grafting: a historical cohort study
Source: J Cardiothorac Surg. 2018 Nov 19;13:118. doi: 10.1186/s13019-018-0798-2 (PMC6245532; doi:10.1186/s13019-018-0798-2)
Supplement: Supplementary file 1 — Table S1. Reference scores for SF-12 health related quality of life. Table S1 shows reference scores for the Short Form 12 health related quality of life questionnaire in the Dutch population. (PDF 84 kb) [file 13019_2018_798_MOESM1_ESM.pdf]

Supplementary table 1 Reference scores for SF-12 health related quality of life (26).

Age group	Physical HRQL	Mental HRQL
Overall	50.6 ± 9.2	50.2 ± 9.2
50 – 59 years	50.5 ± 9.5	50.7 ± 9.0
60 – 69 years	50.5 ± 8.2	51.1 ± 8.7
70 – 79 years	44.4 ± 11.2	49.5 ± 8.5
