# Supplementary material for: Preoperative determinants of quality of life a year after coronary artery bypass grafting: a historical cohort study
Source: J Cardiothorac Surg. 2018 Nov 19;13:118. doi: 10.1186/s13019-018-0798-2 (PMC6245532; doi:10.1186/s13019-018-0798-2)
Supplement: Supplementary file 2 — Table S2. Baseline characteristics for patients with and without postoperative SF-12. Data are presented as mean (±standard deviation), medians (interquartile range) or frequencies (%). * Intraoperative packed red blood cell transfusion ** Blood loss at 24 h after surgery. Table S2 shows baseline characteristics for excluded patients with missing postoperative Short Form 12 questionnaires. (PDF 32 kb) [file 13019_2018_798_MOESM2_ESM.pdf]

Supplementary table 2. Baseline characteristics for patients with and without postoperative SF-12

Demographics	With pre- and postoperative SF-12 N=658	With missing SF-12 N=1442	P value
Age (yr)	65.5 ± 9.0	66.3 ± 10.2	0.003
Male gender	542 (82.4%)	1110 (77.0%)	0.005
Body mass index (kg/m2)	26.8 (24.6-29.8)	27.0 (24.6-29.9)	0.736
Hypertension	338 (51.4%)	761 (52.8%)	0.550
Atrial fibrillation	26 (4.0%)	44 (3.1%)	0.287
Diabetes Mellitus	133 (20.2%)	376 (26.1%)	0.004
Stroke	54 (8.2%)	129 (8.9%)	0.577
Pulmonary disease	50 (7.6%)	122 (8.5%)	0.504
Peripheral vascular disease	68 (10.3%)	166 (11.5%)	0.426
Unstable Angina	65 (9.9%)	177 (12.3%)	0.111
Myocardial infarction	75 (11.4%)	222 (15.4%)	0.015
Left ventricular ejection fraction			0.002
Normal (>50%)	546 (83.0%)	1074 (74.5%)	
Moderate .(30-50%)	94 (14.3%)	293 (20.3%)	
Poor (<30%)	18 (2.7%)	53 (3.7%)	
EuroSCORE	3 (1-4)	3 (2-5)	<0.001
Preoperative creatinine (umol/l)	82 (73-95)	84 (73-99)	0.127
Preoperative hemoglobin (mmol/l)	8.8 (8.2-9.3)	8.6 (7.9-9.2)	<0.001
Surgical characteristics			
Duration of surgery (min)	189 (163-226)	193 (161-228)	0.419
Extra corporeal circulation time (min)	83 (68-101)	84 (68-103)	0.783
Mini extra corporeal circulation	485 (73.7%)	990 (68.7%)	0.019
Use of internal mammary artery	626 (95.1%)	1359 (94.2%)	0.404

Packed red blood cell transfusion*	54 (8.2%)	193 (13.4%)	0.001
Blood loss (ml)**	660 (519-850)	700 (530-938)	0.002

Data are presented as mean (±standard deviation), medians (interquartile range) or frequencies (%).

* Intraoperative packed red blood cell transfusion

** Blood loss at 24 hours after surgery
